# Characterization of a Pre-Clinical Mini-Pig Model of Scaphoid Non-Union 

**DOI:** 10.3390/jfb6020407

**Published:** 2015-06-16

**Authors:** Dominique Andre Behrends, Leticia Khendek, Chan Gao, Nadia Zayed, Janet Elizabeth Henderson, Paul Andre Martineau

**Affiliations:** 1 Bone Engineering Labs, Research Institute-McGill University Health Centre, Montreal General Hospital, 1650 Cedar Ave, Montreal, Quebec H3G 1A4, Canada; E-Mails: domi.behrends@gmail.com (D.A.B.); leticia.khendek@mail.mcgill.ca (L.K.); chan.gao@mail.mcgill.ca (C.G.); nadia.zayed@yahoo.ca (N.Z.); paul.martineau@mcgill.ca (P.A.M.); 2 Experimental Surgery, Faculty of Medicine, Montreal General Hospital, 1650 Cedar Ave, Montreal, Quebec H3G 1A4, Canada; 3 Experimental Medicine, Faculty of Medicine, McGill University, Room 101, 1110 Pins Avenue West, Montreal, Quebec H3A 1A3, Canada

**Keywords:** dense collagen gel, pre-clinical model, headless titanium screw, quantitative micro CT

## Abstract

A fractured scaphoid is a common disabling injury that is frequently complicated by non-union. The treatment of non-union remains challenging because of the scaphoid’s small size and delicate blood supply. Large animal models are the most reliable method to evaluate the efficacy of new treatment modalities before their translation into clinical practice. The goal of this study was to model a human scaphoid fracture complicated by non-union in Yucatan mini-pigs. Imaging and perfusion studies were used to confirm that the anatomy and blood supply of the radiocarpal bone in mini-pigs were similar to the human scaphoid. A 3 mm osteotomy of the radiocarpal bone was generated and treated with immediate fixation or filled with a dense collagen gel followed by delayed fixation. Bone healing was assessed using quantitative micro computed tomography and histology. With immediate fixation, the osteotomy site was filled with new bone across its whole length resulting in complete bridging. The dense collagen gel, previously shown to impede neo-vascularization, followed by delayed fixation resulted in impaired bridging with less bone of lower quality. This model is an appropriate, easily reproducible model for the evaluation of novel approaches for the repair of human scaphoid fractures.

## 1. Introduction

The scaphoid bone at the base of the thumb and the distal radius are the two most commonly fractured bones in the upper extremity [1]. The incidence of scaphoid fractures ranges from 26 to 39 cases per 100,000 individuals each year, with young men between the ages of 25–35 years most commonly affected [2]. The diagnosis of scaphoid fracture remains challenging as its clinical presentation is indistinguishable from a wrist sprain and is not always apparent on plain radiographs [3]. The problems associated with clinical presentation of scaphoid fractures can delay treatment, thus significantly increasing the risk of non-union, which occurs in 10% of cases. Another factor contributing to the high incidence of scaphoid non-union, particularly the proximal pole, is the relatively tenuous blood supply [4]. The major vascular supply to the human scaphoid arises from the superficial palmar branch of the radial artery in the wrist. Vessels enter the scaphoid via vascular foramina located on the volar and dorsal aspects [5]. Whereas the distal scaphoid has direct arterial inflow, the proximal pole is supplied solely by intra-osseous retrograde flow, which is susceptible to interruption in the case of a fracture. Taken together with its small anatomical size, the relatively sparse blood supply to the scaphoid presents a significant challenge to fracture healing. Delayed or inadequate immobilization of the fractured scaphoid leads to non-union and deformity associated with decreased grip strength and wrist motion, degenerative radiocarpal osteoarthritis, and ultimately collapse of the wrist [6,7]. Development of a relevant and reliable pre-clinical model to examine therapeutic interventions to promote scaphoid repair in the setting of delayed treatment is therefore of critical importance to reduce disability in young, physically active individuals. 

Animal species that have been used to examine bone healing in response to novel therapeutics like scaffolds, stem cells and biologics include rodents, rabbit, dog, sheep, goat, horses, and miniature swine. Native or genetically modified rodents are most commonly used during the initial stages of investigation to determine the physiological role of identified molecules *in vivo*. However, larger species with bone properties that resemble those of humans are required for definitive pre-clinical testing. Long bone fractures are commonly modeled in rats and rabbits, whereas dogs and sheep have historically been the most popular preclinical large animal model [8]. The composition and architecture of canine long bones most closely resemble those of humans, and the large size allows for intra-subject comparisons of novel therapeutic strategies, including orthopaedic devices [9]. 

In contrast to the variable genetic background and ethical stigma associated with the use of dogs in bio-medical research, purpose-bred mini-pigs are available from inbred colonies maintained in commercial (e.g., Sinclair bio-resources, MO, USA) and academic (e.g., Memorial University, Newfoundland, Canada) facilities. The mini-pig skeletal macrostructure, physiology and lamellar micro-structure closely resemble those of human bone, although the trabecular network is marginally denser [10,11,12]. Adult mini-pig bone undergoes continuous turnover at a rate similar to that seen in human bone [13], and its metabolism is susceptible to withdrawal of hormones such as that seen in aging or induced by oophorectomy [14]. More relevant to this study, the mini-pig radiocarpal bone is similar in size, shape and location to the scaphoid equivalent in humans. The nutrient vessels enter the dorsal pole of the radiocarpal bone and supply the volar pole by intra-osseous blood flow. Thus, the dorsal and volar poles of the radiocarpal bone in mini-pig are analogous to the distal and proximal pole respectively of the scaphoid in humans. The goal of this study was to develop a simple, consistent and anatomically relevant model of scaphoid non-union for future pre-clinical evaluation of novel therapeutic interventions to promote bone regeneration and repair. 

## 2. Materials and Methods

### 2.1. Mini-Pig Purchase and Maintenance

Young adult Yucatan miniature swine age 11 month (±1 month) with a mean weight of 39 kg (±2 kg) were purchased (Memorial University colony, Newfoundland, Canada) and shipped air freight to our large animal facility (Montreal General Hospital Site, RI-MUHC, Montreal, Quebec, Canada) where they were maintained for up to six months. Animals were housed in single stalls and fed a standard mini-pig diet (Teklad, Harlan, Montreal, Canada) twice a day with free access to water for the duration of the study. All animal procedures were performed in strict accordance with a protocol approved by the McGill Facility Animal Care Committee, in keeping with the guidelines of the Canada Council on Animal Care.

### 2.2. Vascular Perfusion with Contrast Agent

To minimize the number of live animals used for this study, we performed vascular mapping of the forelimb of a domestic piglet used for experimental surgical techniques. The 35 kg piglet was euthanized using an IV infusion of pentobarbital, the brachial arteries exposed to insert 14 gauge catheters and the vasculature flushed with 0.9% saline containing 100 U/mL heparin. The cadaver was then perfused with a radio-opaque barium sulphate (BaSO_4_) solution composed of 50% BaSO_4_ in a 5% gelatin solution containing 0.25% and 0.75% D-sorbitol in 0.9% saline. The proximal and distal carpal bones were dissected *en bloc* with the surrounding soft tissues and fixed as above for micro CT analyses.

### 2.3. Bilateral Mini-Pig Surgery

#### 2.3.1. Preparation of Spacers for Delayed Fixation 

Dense collagen gels, shown previously to block vascular ingrowth and repair of a large defect in the femoral diaphysis of a mouse [15], were prepared on the morning of surgery to fill the 3 mm gap for delayed fixation. In brief, a solution of 2.2 mg/mL rat tail Type I collagen (First Link, West Midlands, UK) was diluted to 1.95 mg/mL with Dulbecco’s Modified Eagle’s Medium (DMEM) (Sigma-Aldrich, Oakville, ON, Canada), and the pH adjusted to 7.5 with NaOH. 200 μL of alpha MEM (Sigma-Aldrich) was added to each 800 μL of pH adjusted collagen solution. The solution was then transferred into one well of a 24-well plate, incubated for 30 min at 37 °C for gelation, and was subjected to unconfined compression under a load of 1.4 kPa for 3 min to form a dense gel with an estimated collagen fibrillar density of 4.8 wt %.

#### 2.3.2. Mini-Pig Sedation and Anesthesia

After 2 weeks acclimatization the animals were fasted for 6–12 h prior to receiving pre-operative sedation intramuscular (IM) with a cocktail containing 0.25 mg/kg butorphanol: 0.19 mg/kg acepromezine: 0.009 mg/kg glycopyrrolate followed by IM injections of 14 mg/kg ketamine and 0.05 mg/kg atropine. After induction of anesthesia with 30 mg/kg sodium pentobarbital, the mini-pigs were intubated to maintain anesthesia with 2%–3% isoflurane delivered with O_2_ at 2 L/min for the duration of surgery. A single dose of 25 mg/kg cephalexin was administered and the animals hydrated during surgery with Ringer’s lactate solution administered via an ear vein.

#### 2.3.3. Generation of LEFT 3 mm Defect with Immediate Fixation

Animals were placed in a right lateral recumbent position and the LEFT forefoot prepared for aseptic surgery by cleaning the skin with iodine and routine sterile draping. The radiocarpal bone was identified by fluoroscopy and the incision site was identified with a sterile marker. The radiocarpal bone was located directly under the skin and was not covered by any muscle, tendon, nerve or vessels. A longitudinal incision was centered over the radiocarpal bone starting 1 cm proximal to the joint line with the radius and extending 5 cm distal. The joint capsule was elevated over the waist and volar aspect of the radiocarpal bone to ensure perfect visualization of the osteotomy site and insertion of the fixation screw. The volar and dorsal aspects of the osteotomy site were marked and an oscillating saw used to transect the full thickness of the bone under constant cooling with Ringer’s solution. A double transverse osteotomy was performed to generate a 3 mm gap. The 3 mm segment of bone was removed while the dorsal and volar fragments were held in place by the native ligaments attachments to the bone. Under fluoroscopic guidance, the 3 mm gap was fixed temporarily with a 0.045 inch guide wire (Acutrak, Hillsboro, OR, USA) while an Acutrak-2 mini conical drill with cannula was used to generate a screw hole. A 20 mm threaded titanium headless compression screw (Synthes, Mississauga, ON, Canada), similar to that used in the clinic, was then used to fix the proximal and distal bone fragments at a constant 3 mm distance from one another. The joint capsule, subcutaneous layer and skin were then closed separately.

#### 2.3.4. Generation of RIGHT 3 mm Defect with Delayed Fixation

After closing the wound on the left forelimb the animal was positioned in a left recumbent position and the RIGHT forelimb prepared for sterile surgery as described above. A 3 mm osteotomy was performed as above but instead of immediate stable fixation with a headless compression screw, a dense collagen spacer prepared as in 2.3.1 was packed into the osteotomy site and the wound layers were closed without providing any additional fixation. After recovery from anesthesia the animals were given post-operative care as described below and allowed free range of motion for seven weeks when the animals underwent a second surgery on the RIGHT side only. The radiocarpal bone was accessed through the previous incision site and the collagen spacer and surrounding fibrous scar tissue removed by surgical dissection. The proximal and distal bone fragments then underwent rigid fixation with a 20 mm headless compression screw as described in 2.3.3. There was no further intervention on the left side.

#### 2.3.5. Mini-Pig Postoperative Management and Care

On recovery from the anesthetic mini-pigs were allowed free range of motion and un-restricted access to food and water for the duration of the experiment. Animals were monitored daily for signs of inflammation, lameness and for general well-being by an experienced animal care technician and the veterinarian. Post-operative analgesia was achieved with a 75 mg fentanyl dermal patch applied to the shoulder and IM administration of 5–20 µg/kg buprenorphine every 6–12 h for three days. An amount of 10 mg/kg/day antibiotic was administered with food for 10 days post-operation. Latero-medial and antero-posterior X-rays and CT images, at 0.2 mm resolution, were captured on anesthetized mini-pigs using a Fidex, Animage instrument (Pleasanton, CA, USA) immediately after primary and secondary surgical procedures, and at the time of euthanasia 19 weeks after the first procedure. IV injection of 30 mg/kg calcein was administered eight and three days prior to euthanasia to label active bone forming surfaces. Mini-pigs were euthanized by injection of a 10× overdose of sodium pentobarbital and potassium chloride. The proximal and distal carpal bones were dissected *en bloc* with the surrounding soft tissue, fixed overnight at 4 °C in fresh 4% paraformaldehyde and rinsed ×3 with PBS before micro CT scanning (see below).

### 2.4. Post-Mortem Analyses

#### 2.4.1. Micro CT Visualization of Vasculature

A high resolution Skyscan 1172 micro CT instrument (Soquelec, Montreal, QC, Canada) was used at a voltage of 80 kV and power of 10 W, with 0.5 mm aluminum and copper filters to reduce beam hardening. Scans were captured at 13 µm spatial resolution, reconstructed into 2D and 3D images with NRecon and DataViewer software supplied with the instrument. 

#### 2.4.2. Micro CT Analysis of Bone Repair

All other terminal imaging and histological analyses were performed as described previously for rodent models [16]. The carpal bones were scanned and the images reconstructed as described above and the dataset quantified using CTAn, and CTVol software. Quantitative data on bone mass and architecture was evaluated in a ring-shaped regions of interest (ROI) extending 1 mm from the screw. 

#### 2.4.3. Histological Analysis of Bone Repair

After micro CT scanning the specimens were embedded in polymethylmethacrylate at low temperature to preserve enzyme activity in osteoclasts. 60 µm thick sections were cut in the coronal plane using a Leica SP1600 saw microtome (Leica Microsystems, Concord, ON, Canada). Sections were stained with 2% Alizarin Red to identify bone mineral and counterstained with 1% methylene blue. Tartrate-resistant acid phosphatase activity (TRAP) in osteoclasts was identified by staining adjacent sections with naphthol AS-TR phosphate, sodium nitrite, sodium tartrate, and pararosaniline hydrochloride in acetate buffer at pH 5.0 and counterstaining with fast green. Active bone forming surfaces in the ROI were identified by fluorescence microscopy of the calcein labeling, which was correlated with bone morphology on the same sections stained with 0.2% toluidine blue. Images were analyzed with a Zeiss Axio Imager M2 (Jena, Germany). 

## 3. Results and Discussion

### 3.1. Mapping Scaphoid Bone Morphology and Vascular Supply 

**Figure 1:**
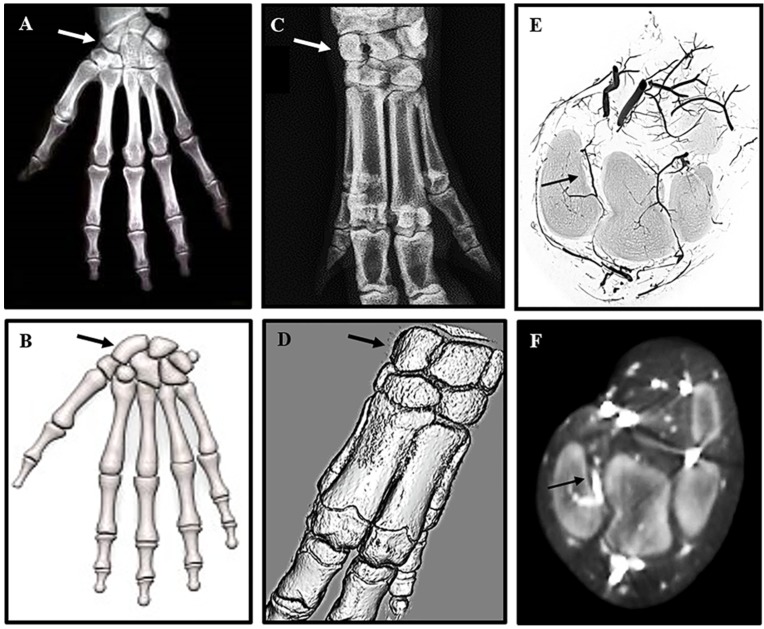
High resolution radiographs comparing human scaphoid and mini-pig radiocarpal bone: The position of the scaphoid bone in the human wrist (**A**,**B**), and the corresponding radiocarpal bone in a porcine forelimb (**C**,**D**) are marked by arrows in 2D X-rays (A,C) and 3D models (B,D). For contrast radiography pigs were euthanized, and the forelimbs perfused immediately with BaSO_4_ via the axillary artery. The carpal bones were scanned at high resolution on a Skyscan 1172 *ex vivo* instrument (**E**) and on a Fidex Animage *in vivo* instrument (**F**). The position and size of the pig radiocarpal bone closely resemble those of the human scaphoid bone, but differ in orientation of the longitudinal axis. As with the human scaphoid, the pig radiocarpal bone has a limited blood supply to the volar pole indicated by arrows on the 3D reconstructions (E,F).

Radiologic images and 3D representations (Figure 1) of the adult human hand (Figure 1A,B) and mini-pig forelimb (Figure 1C,D) reveal a remarkable similarity in anatomy, particularly of the complex structure of the carpal bones in the wrist. The human scaphoid corresponds to the mini-pig radiocarpal bone (arrows Figure 1A–D), which is located between the radius and the metacarpal bones. BaSO_4_ contrast agent injected into the axillary artery to visualize the vascular feed to the radiocarpal (Figure 1E,F) confirmed the same organization of retrograde blood supply as seen in the human scaphoid. Reconstruction of high resolution micro CT images (Figure 1E) confirmed the position of the supply artery, originating from the large vessels on the volar side of the wrist and entering the dorsal aspect of the radiocarpal bone. The intraosseous vessel then runs from dorsal to volar analogous to the retrograde flow of the radial artery blood supply in the human scaphoid, leaving the volar pole vulnerable to avascular necrosis. The course of the blood vessels was confirmed on transaxial CT images captured with the Fidex Animage CT scanner (Figure 1F). The radiocarpal bone was, thus, shown to resemble the human scaphoid bone and the mini-pig identified as an appropriate pre-clinical model to replicate fracture non-union of the human scaphoid to develop improved therapeutic interventions for repair.

### 3.2. Surgical Outcomes and Bone Repair 

**Figure 2:**
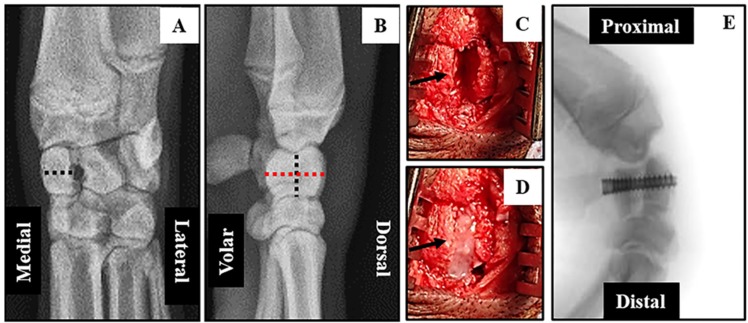
Surgical intervention to generate radiocarpal non-union: X-rays of the forearms of anesthetized mini-pigs were obtained in the coronal (**A**) and sagittal (**B**) planes to measure the dimensions (dotted lines) of the radiocarpal bones prior to surgery. Medial-Lateral = 7.4 mm; Volar-Dorsal = 17.5 mm and Proximal-Distal 10.5 mm. 3 mm osteotomies (**C** arrow) were performed through the mid-radiocarpal bone of both forelimbs. The volar and dorsal pieces of the **LEFT** radiocarpal (control) were fixed immediately and a dense-collagen spacer (**D** arrow) inserted in the gap of the **RIGHT** radiocarpal (non-union). After 7 weeks of healing the LEFT radiocarpal had no further intervention while the spacer in the RIGHT radiocarpal was removed, the volar and dorsal segments re-apposed and fixed with a screw (**E**). The mini-pigs were left for an addition 12 weeks before termination of the experiment.

High resolution X-rays taken pre-operatively were used to confirm the dimensions of each radiocarpal bone prior to surgical intervention, and post-operative to ensure adequate fixation of the defect (Figure 2). 

**Figure 3:**
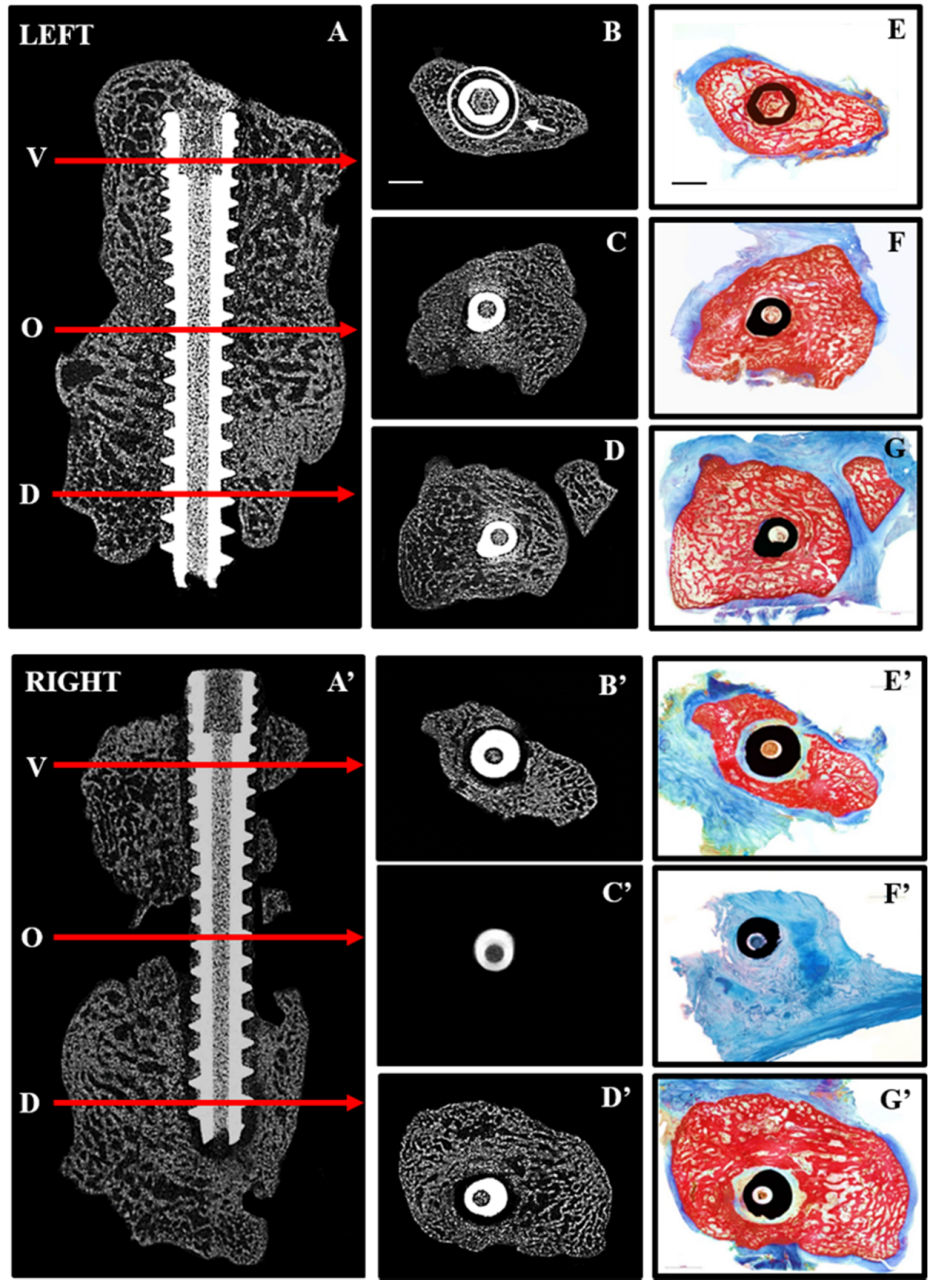
Repair after immediate (**LEFT**) and delayed (**RIGHT**) fixation: 2D micro CT images are shown in the sagittal plane (**A**,**A**’) indicating the position along the screw (arrows) at which transaxial 2D images are shown for the volar (**V**,**B**,**B**’), osteotomy site (**O**,**C**,**C**’) and dorsal (**D**,**D**,**D**’) aspects of the radiocarpal bones. Corresponding histological sections from the same levels (**E**–**G**,**E**’–**G**’) were stained with Alizarin red and Methylene blue to distinguish mineralized (red) from soft (blue) tissue. Complete bridging occurred with immediate fixation (**A**,**C**) whereas little bone was seen in the osteotomy site (**A**’,**C**’) with delayed fixation. Histological sections confirmed the presence of bone in the osteotomy site with immediate fixation (**F**) and its absence with delayed fixation (**F**’). Arrow (**B**) points to ROI used for quantitative micro CT analyses and scale bars (**B**,**E**) represents 2 mm.

The time taken to anesthetize the animals and perform bilateral surgical interventions on each of three mini-pigs varied between 80 min and 110 min. Surgeries were uneventful except for a partial fracture of the volar aspect of the left radiocarpal at the time of screw insertion in the first animal operated. This bone was subsequently excluded from all post-mortem analyses leaving only two animals for direct comparison of bone repair after immediate fixation (IF) and three for delayed fixation (DF). All mini-pigs were fully ambulatory within 48 hours post-operation and had no visible signs of infection or other complications after one week. Micro CT and un-decalcified histological analyses (Figure 3) clearly demonstrated mal-union of the right radiocarpal bone at 19 weeks post-operative. The dorsal and volar segments were rigidly fixed at seven weeks after removal of the dense collagen spacer.

**Figure 4:**
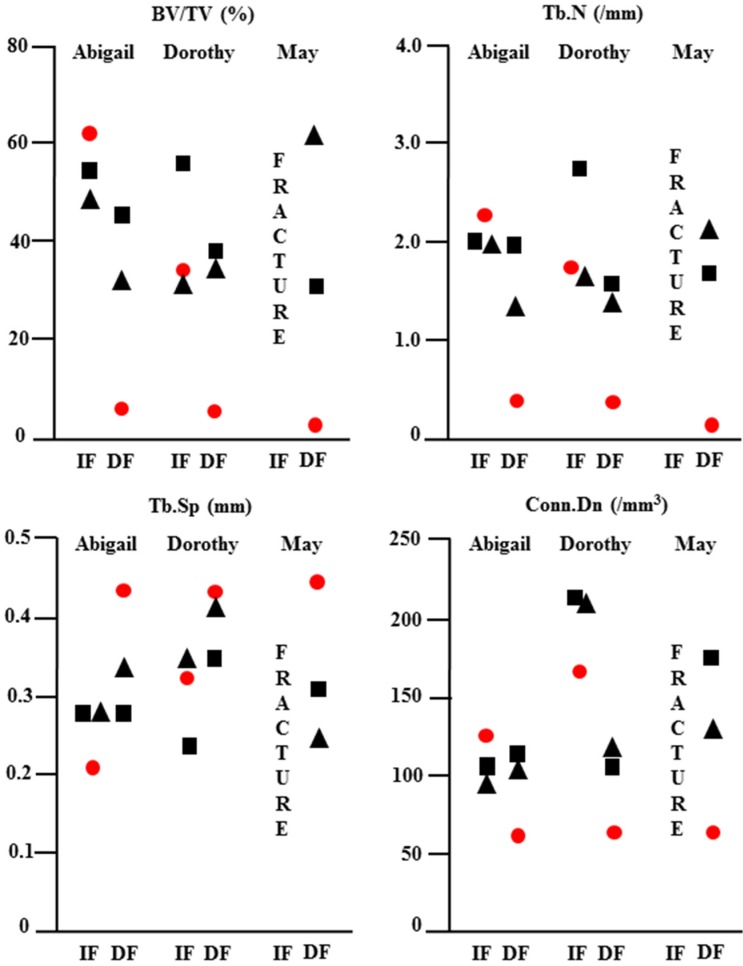
Quantitative micro CT analysis of repair after immediate (IF) and delayed (DF) fixation: The osteotomy site and adjacent bone in the LEFT (IF) and RIGHT (DF) radiocarpal bones of minipigs Abigail, Dorothy and May were scanned at 19 weeks post-operative and the data analysed using CtAn software. Quantitative data for bone volume/tissue volume (BV/TV), trabecular number (Tb.N), trabecular separation (Tb.Sp) and trabecular connectivity (Conn.Dn) are shown for the dorsal (square), osteotomy site (circle) and volar (triangle) aspects of the bone. Delayed fixation resulted in significantly less bone with poor quality at the osteotomy site in all animals.

In contrast to immediate fixation (IF) (Figure 3A–G), where bone was seen adjacent to the screw along its entire length, delayed fixation (DF) resulted in soft tissue along the length of the screw (Figure 3A’–G’). This is most noticeable on the histological sections where bone is red and soft tissue blue. Quantitative micro CT (Figure 4) revealed significantly less bone (BV/TV) adjacent to the screw at the osteotomy site (white circle in Figure 3B) with DF compared with IF. 

**Figure 5:**
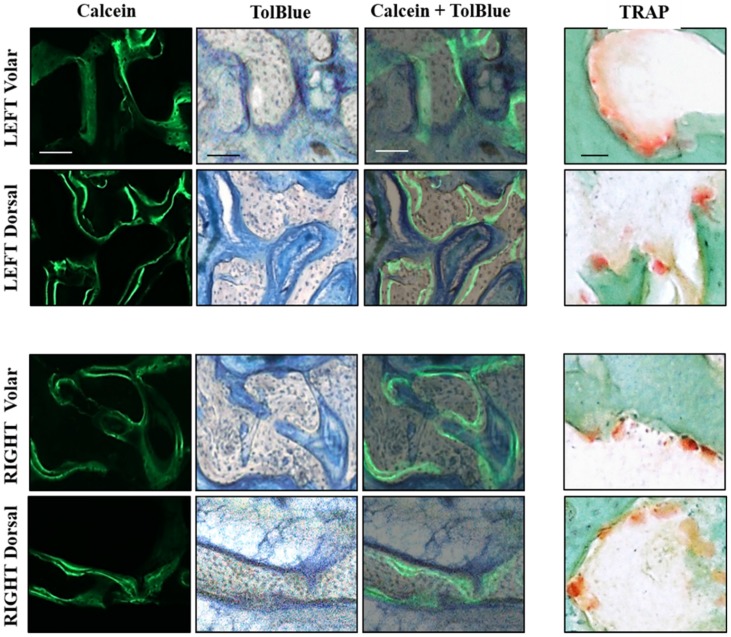
Histological analysis of volar and dorsal aspects of radiocarpal bone: 60 µm sections were cut with a saw microtome from the volar and dorsal aspects of the LEFT (upper) and RIGHT (lower) radiocarpal bones, adjacent to those stained with Alizarin red in Figure 4. The sections were first visualized under fluorescence microscopy to capture images of calcein labeling before staining with TolBlue to distinguish mineralized (grey) from soft (blue) tissue. The images were then super-imposed (Calcein + TolBlue) to identify active bone-forming surfaces. Adjacent sections were stained with TRAP and Fast Green to identify osteoclasts on surfaces undergoing resorption. No differences were seen in any staining between the Volar and Dorsal aspects or between LEFT and RIGHT radiocarpal bones. Scale bars represent 100 µm for Calcein and TolBlue images and 50 µm for TRAP images.

This trend was reflected by a significant reduction in the number (Tb.N) and connectivity (Conn.Dn) of trabeculae at the osteotomy site with DF. Histological analyses of bone formation and resorption are shown in 60 µm thick bone sections from the dorsal and volar aspects of left (IF) and right (DF) radiocarpal bones (Figure 5). Dynamic labeling of bone formation with calcein, and staining of bone resorbing osteoclasts with TRAP, showed no difference between dorsal and volar aspects or RIGHT (IF) and LEFT (DF) specimens.

### 3.3. Discussion

The diagnosis and treatment of scaphoid fractures remains a significant challenge to orthopedic surgeons. Conventional radiography is often insufficient to make a definitive immediate diagnosis, whereas CT scan and MRI scans are often not obtained initially by the clinician without a strong index of suspicion for a suspected scaphoid fracture. In fact, many patients do not even present early to the hospital or clinic, believing they have only sustained a wrist sprain. Therefore, significant delays in treatment are common in patients with scaphoid fractures. Diagnostic capabilities have improved using gadolinium-enhanced MRI imaging in order to evaluate the vascular feed and potential viability of the proximal pole following acute scaphoid fracture [17]. However, one still requires a clinical suspicion of fracture in order to obtain the test. 

Non-displaced fractures with viable proximal and distal fragments can be treated by a prolonged period of immobilization in a cast, thereby avoiding surgery-related complications. However, prolonged immobilization leads to muscle atrophy and stiffness that may require an extended period of physiotherapy to achieve pre-injury function. Since the majority of scaphoid fractures occur in young patients, the need for prolonged immobilization can also have significant consequences related to time required off work. Hence, there has been a recent trend to expedite return to full early activities by treatment with immediate surgical fixation, even for non-displaced fractures, although displacement is commonplace [18,19]. The mainstay of treatment for scaphoid fractures is therefore early surgical fixation with a headless compression screw, similar to that used in the current study. 

Due to delays in presentation, the low diagnostic yield of routine imaging combined with the inefficiency of immobilization, many patients present with pain and reduced mobility of the wrist arising from an established scaphoid non-union. Non-union of scaphoid fractures leads to a progressive pattern of degeneration of the wrist joint known as scaphoid non-union advanced collapse. Therefore, it is important to treat scaphoid non-union to avoid the development of degenerative changes in the wrist. The management of scaphoid non-union is challenging and complex and must be addressed surgically with internal fixation in a similar manner to the treatment of acute fractures. However, the healing potential of a non-union is significantly reduced compared with acute fractures and can typically require bone grafting and/or revascularization procedures to improve the biology of the healing milieu. In addition, scaphoid non-union also commonly leads to bone loss and deformity, requiring the use of structural bone grafts. All of these adjunct procedures add to the surgical morbidity associated with the treatment of scaphoid non-union, compared with the treatment of an acute fracture. Despite the use of all these adjunct techniques, the healing rate for scaphoid non-union remains lower than for acute fracture. The significant challenges and morbidity involved in the treatment of scaphoid non-union therefore necessitates the development of novel approaches that will simplify the surgical intervention and reduce morbidity. This is best accomplished in an appropriate, reproducible, pre-clinical large animal model, such as the one we have described [20].

Domestic pigs and miniature swine have been traditionally used to investigate diagnostic and therapeutic interventions for circulatory disorders like avascular necrosis of the femoral head [21,22,23]. Using contrast agent and high resolution micro CT imaging we showed in the current study that the vascular supply to the radiocarpal bone in swine is similar to that in humans. The limited blood supply to the distal pole can be compromised by fracture. In addition, inadequate immobilization after fracture, as in the case of the RIGHT radiocarpal bones in our study leads to the reproducible creation of a radiocarpal non-union. The presence of fibrous tissue at the osteotomy site, 12 weeks after DF, indicated a Type DI established non-union according to the Herbert classification [24,25]. 

An ultra-rapid engineering approach using unconfined compression of hydrated type I collagen gels was developed a decade ago to manufacture biomimetic materials for bone tissue engineering [26]. The approach allowed us to generate a dense collagen material after plastic compression with mechanical properties similar to osteoid. Studies in the laboratories of colleagues have investigated the enhanced *ex vivo* biomineralization properties of dense collagen gels, alone [27] or in composite materials with silk fibroin [28]. However, in our previous work using a mouse model of bone healing, it was shown that the dense collagen material formed an effective barrier to *in vivo* bone repair [15] in the absence of an angiogenic agent like VEGF. For this reason we hypothesized that the dense collagen gels would also inhibit neo-vascularization in the 3 mm defect during the early phase of bone regeneration and effectively inhibit repair of the defect in the longer term. In the current study, the absence of bone bridging in the right radiocarpal bones demonstrates that the dense collagen spacer, when inserted in the fracture gap for seven weeks prior to delayed fixation, served as an effective method to promote the reproducible creation of non-union of the radiocarpal bone that can be used to investigate novel therapeutic interventions and/or fixation hardware. For example, this mini-pig non-union model will be used to investigate the *in vivo* efficacy of a patented porous titanium screw for improved scaphoid fixation. In recent work, the biomechanical properties and insertion profiles of the screw were shown to compare favorably with those of the commercially available solid titanium screws [29]. 

## 4. Conclusions 

The current study using a dense collagen spacer and delayed fixation of a 3 mm segmental defect in the mini-pig radio-carpal bone represents an effective, reproducible and clinically relevant model of human scaphoid non-union. The model lends itself to the development of novel interventions to promote re-vascularization and expedite bone regeneration that will restore pre-injury wrist function to otherwise healthy young individuals.
